# Type 1 Diabetes: Interferons and the Aftermath of Pancreatic Beta-Cell Enteroviral Infection

**DOI:** 10.3390/microorganisms8091419

**Published:** 2020-09-15

**Authors:** Pouria Akhbari, Sarah J Richardson, Noel G Morgan

**Affiliations:** Islet Biology Exeter (IBEx), Institute of Biomedical and Clinical Science, College of Medicine and Health, University of Exeter, Exeter EX2 5DW, UK; s.richardson@exeter.ac.uk (S.J.R.); n.g.morgan@exeter.ac.uk (N.G.M.)

**Keywords:** enterovirus, Interferon Stimulated Genes (ISG), Endoplasmic Reticulum (ER) stress, apoptosis, innate immunity, autoimmune disease

## Abstract

Enteroviruses (EVs) have long been implicated in the pathogenesis of type 1 diabetes (T1D), and accumulating evidence has associated virus-induced autoimmunity with the loss of pancreatic beta cells in T1D. Inflammatory cytokines including interferons (IFN) form a primary line of defence against viral infections, and their chronic elevation is a hallmark feature of many autoimmune diseases. IFNs play a key role in activating and regulating innate and adaptive immune responses, and to do so they modulate the expression of networks of genes and transcription factors known generically as IFN stimulated genes (ISGs). ISGs in turn modulate critical cellular processes ranging from cellular metabolism and growth regulation to endoplasmic reticulum (ER) stress and apoptosis. More recent studies have revealed that IFNs also modulate gene expression at an epigenetic as well as post-transcriptional and post-translational levels. As such, IFNs form a key link connecting the various genetic, environmental and immunological factors involved in the initiation and progression of T1D. Therefore, gaining an improved understanding of the mechanisms by which IFNs modulate beta cell function and survival is crucial in explaining the pathogenesis of virally-induced T1D. This should provide the means to prevent, decelerate or even reverse beta cell impairment.

## 1. Introduction

Type 1 diabetes (T1D) is a chronic autoimmune disease characterised by reduced insulin production and caused by a specific immune-mediated loss of insulin producing beta cells [1,2]. Aetiologically, the initiation and progression of T1D occurs as a consequence of a complex interplay between genetic, immunological and environmental factors. A number of genes and their variants have been linked to risk of T1D, the majority of which are associated with immune responses or modulation of immune pathways [3]. From an environmental perspective, factors such as diet, gut microbiota and infections have been indicated as triggers of T1D [2]. Among the infectious agents, many studies have implicated infection by enteroviruses (EVs), but it has not been established which EV serotypes (among the more than 100 known) are involved. The available evidence supports infection by various strains of the Coxsackievirus B (CVB) and some Echoviruses [4,5,6,7,8,9,10,11,12,13,14,15,16,17,18,19,20,21], but various other viruses including rubella, mumps, reoviruses, parvoviruses and cytomegalovirus have also been linked to T1D [22,23]. However, evidence for the implication of the latter viruses in T1D has been challenged on the basis of a weak link and/or irreproducibility of the association [24]. In addition, it has been noted that despite the eradication of mumps and rubella viruses following large-scale vaccination programmes in many countries, the incidence of autoimmune diseases such as T1D has risen, arguing against this and suggesting that their lack, rather than their presence, may have contributed to increasing incidence of T1D [25]. Nevertheless, EVs are the most commonly identified (and studied) viruses in epidemiological, clinical and histopathology studies of T1D [26]. EVs display a tropism for beta cells and expression of the Coxsackie and adenovirus receptor (CAR, CXADR), one of the key entry receptors for the CVB strains, has been demonstrated in beta cells [27,28], suggesting that systemic infections might culminate in the entry of EVs into these cells. In support of this, both enteroviral RNA and the viral capsid protein 1 (VP1), have been detected at low levels, in the pancreas of people with type 1 diabetes at a higher frequency than in control subjects [29]. Such evidence has been interpreted as evidence of the persistence of a low grade infection in beta cells [7,30]. A temporal association between EV infection and seroconversion to islet cell antibody (ICA) immunopositivity has also been described [31], and a meta-analysis of twenty six case control studies concluded that EV infection is significantly linked with autoimmunity and T1D [20]. EV RNA has also been detected in peripheral blood mononuclear cells (PBMC) of recent onset T1D patients at a higher frequency than in nondiabetic control subjects [29,32], and the presence of EV RNA in the serum of patients has been suggested as a risk factor for beta cell autoimmunity and T1D [33].

The development of T1D has been associated with infection by particular CVB serotypes, since, for instance, an increased incidence of T1D was reported following epidemics of CVB4 and CVB5 [34,35], whilst an antibody survey of different European populations suggested that seropositivity for anti-CVB1 antibodies is associated with beta cell autoimmunity and increased risk of T1D [36,37]. The chronological order of infection with different CVB serotypes may also influence the outcome of the immune response. For example, infection with CVB3 or CVB6 prior to infection with CVB1 may reduce the risk of developing autoantibodies compared to when CVB1 infection occurs first [36]. A more recent large scale study of virome in the stools of children from four countries has shown that prolonged shedding of EV-B is associated with an increased likelihood that islet autoimmunity will develop [38]. Nevertheless, despite the accumulation of a significant volume of evidence over a prolonged period, the demonstration of a causal link between the EV infection of islet cells and the onset of T1D is still to be fully verified [39], and it will require the development of a successful vaccination strategy before the issue is finally resolved.

While numerous clinical, epidemiological and experimental studies have associated viruses with T1D, few studies have investigated the efficacy and immunogenicity of vaccines in relation to T1D. Recent evidence has implicated rotavirus vaccination as a means to reduce the incidence of T1D [40], but the weight of the evidence is still debated [41]. Other studies in mouse models of T1D have shown that mice vaccinated against CVB1 have significantly lower levels of replicating virus both in their blood and in the pancreas and are protected against T1D acceleration when challenged with CVB1 [42,43]. An inactivated hexavalent CVB vaccine induces robust antibody responses against the six known CVB serotypes and has proved safe when administered to mice and nonhuman primates. Importantly, this vaccine prevented T1D in a mouse model highly susceptible to CVB-induced beta cell destruction [44].

It may be argued that a causal link between virus infections and autoimmune T1D should imply a concordant incidence rate between the two. Whilst many studies have implicated virus infections as promoters of T1D (see above), a few other studies are suggestive of an overall protective role for viruses against T1D. For instance, lower prevalence of T1D (and other autoimmune diseases) in equatorial countries compared to northern Europe and north America has been attributed to higher hygiene standards (and consequently lower infection rates) in the latter regions [45]. This notion has been supported by epidemiological studies in migrant communities with an increased incidence rate of T1D in their new country of residence [46,47,48]. The incidence rate of T1D was also varied and inversely correlated with frequency of EV antibodies in several northern and eastern European countries [49,50] even when populations with a similar genetic background, but with different EV infection rates, were compared [51]. Nevertheless, whilst large scale epidemiological studies to prove or disprove this notion are still lacking, it is important to emphasise that viruses, even if causally linked to T1D, are not the sole determinants of beta cell fate. Rather, they are likely to contribute to the onset and/or progression of autoimmune T1D in the presence of other factors such as a predisposing genetic background (which is frequently enriched in specific geographical populations) and an aberrant immune response against pancreatic beta cells.

Despite these ongoing uncertainties, it is well documented that mammalian cells initiate a coordinated early phase autonomous response to viral and bacterial infections, which is mediated by interferons (IFN), especially type I and type III IFNs, and other pro-inflammatory cytokines. This is manifest in various ways, including by the induction of a plethora of IFN stimulated genes (ISGs) [52,53]. IFNs typically impact pancreatic beta cell function and survival by augmenting their intracellular antiviral response mechanisms to mitigate the potentially negative impacts of viral infection. One such example is suppression of CVB3 infection in pancreatic islets pretreated with type I and type III IFNs [54]. However, they can also (perhaps inadvertently) increase the visibility of beta cells to the immune system by, for example, promoting the hyperexpression of human leukocyte antigen class I (HLA-I) molecules, thereby facilitating the presentation of autoantigens to influent immune cells. Consistent with this, histopathological examination of pancreas sections has revealed key inflammatory signatures in the islets of individuals with recent onset T1D. These include an almost universal hyperexpression of HLA-I molecules and the frequent presence of infiltrating cytotoxic CD8+ T lymphocytes, CD4+ T cells, CD20+ B cells and macrophages [55,56]. Viral infections are potent inducers of IFN production, and these appear to play a key role in driving islet inflammation, as well as being potential mediators of apoptosis and beta cell dysfunction. Supportive evidence for the importance of the IFN pathway in the development of T1D further derives from studies demonstrating that aberrant IFN responses triggered either by genetic mutations in key pathways [57] or the activation of components upstream of IFNs [58] are also associated with disease. Collectively, these findings imply that the IFN pathway is important for the development of T1D, regardless of whether the initial trigger is a viral infection, a genetic mutation or the activation of the IFN pathway by other means. In this review, we provide an overview of recent findings which illuminate the actions of IFNs on beta cells to show how these cytokines impact and modulate cellular responses in a bid to regulate cell survival during viral infection.

## 2. IFNs as a Key Link between Environmental and Genetic Risk Factors of Autoimmune T1D

**Figure 1:**
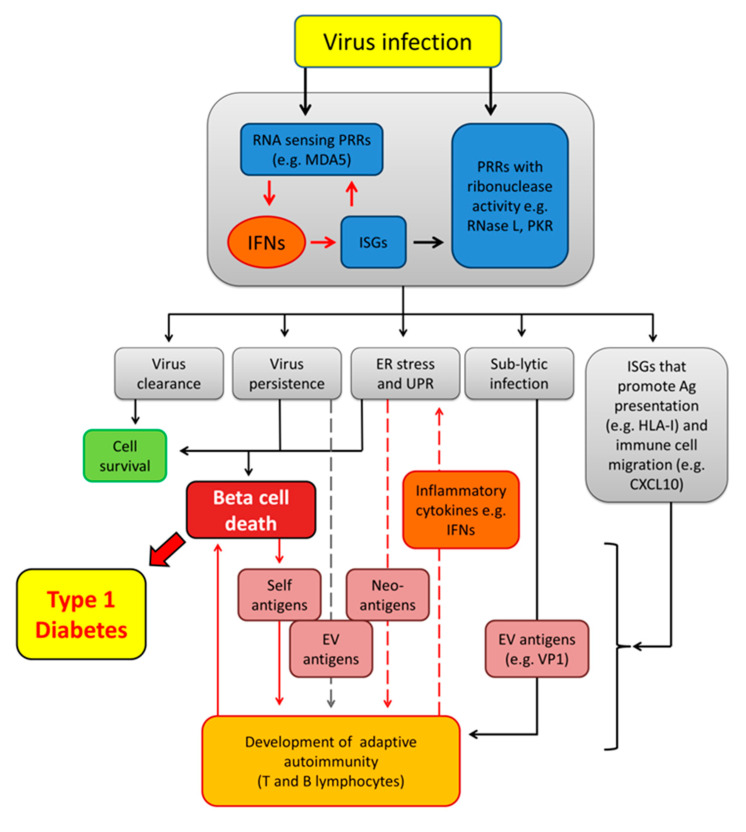
Virus-induced autoimmune T1D. An initial enterovirus (EV) infection activates pattern recognition receptors (PRRs) that induce interferons (IFNs) and interferon stimulated genes (ISGs) as well as PRRs that directly target and cleave viral RNA, driving a collective intrinsic antiviral response that may lead to virus clearance or virus persistence. The surge in IFNs and ISGs promotes Endoplasmic Reticulum (ER) stress and unfolded protein response (UPR) which, alone or in combination with virus infection, may lead to programmed cell death (apoptosis), exposing virus and self-antigens to the immune system. In addition, IFNs promote antigen presentation and immune cell migration by inducing the expression of human leukocyte antigen class I (HLA-I) molecules and chemokines such as C-X-C motif chemokine 10 (CXCL10). Cells with lytic or persistent infection that do not undergo apoptosis may present viral antigens (e.g., VP1) and self-antigens (including neo-antigens induced by IFNs and UPR) to the immune cells (APCs, CD8+ and CD4+ T cells) leading to development of autoantibodies and an adaptive autoimmune response against antigen producing cells. Islet infiltrating immune cells may also contribute to ER stress and UPR in beta cells by releasing inflammatory cytokines such as IFNα and IFNγ. MDA5, melanoma differentiation-associated protein 5; PKR, protein kinase R; VP1, virus capsid protein 1.

As a means to develop antiviral defence mechanisms, mammalian cells have evolved several pattern recognition receptors (PRRs) to sense pathogen associated molecular patterns (PAMP) such as virally encoded nucleic acids [59]. EVs have a small positive sense single-stranded RNA (ssRNA) genome which is copied to generate a double-stranded RNA (dsRNA) intermediate during replication and in chronic stages of infection [60,61,62]. PRRs form part of an upstream ‘early warning’ system which, upon activation, induces IFN secretion to signal the presence of viruses to surrounding cells (Figure 1). Such PRRs include Toll-like receptor 3 (TLR3), melanoma differentiation associated gene 5 (MDA5) and retinoic acid inducible gene I (RIG-I) [59,63]. TLR3 typically resides in the endosomal compartment and is predominantly restricted to immune cells [64]. Consistent with this (and noting that the evidence remains equivocal [65]), we have not been able to detect TLR3 by immunohistochemistry in human beta cells [5]. By contrast, MDA5 and RIG-I both sense dsRNA species present in the cytoplasm of most cells [64]. However, activation of RIG-I is restricted to specific triphosphorylated RNA species having uncapped 5′ termini—a molecular feature normally found in negative sense RNA viruses such as influenza virus [66]—but it is not activated by EV RNAs which lack a 5′ triphosphate [67]. Importantly, activation of MDA5 requires a high molecular weight complex RNA structure composed of both ssRNA and dsRNA [67], and these can be generated during EV infection. Upon engagement with a relevant ligand, MDA5 monomers form an oligomeric filament along the length of the target RNA and an assembly of multiple MDA5 N-terminal caspase recruitment domains (CARDs) then recruit interferon promoter stimulator 1 (IPS-1, also known as MAVS, mitochondrial antiviral signalling protein) [68]. IPS-1 signalling, in turn, engages members of the tumour necrosis factor (TNF) receptor-associated factor (TRAF) family (e.g., TRAF3 and TRAF6) and TRAF family member-associated nuclear factor κB (NF-κB) activator (TANK), leading to increased phosphorylation of NF-κB and both interferon regulatory factor 3 (IRF3) and IRF7. Upon phosphorylation, these proteins translocate to the nucleus and induce expression of IFN type I and other potentially pro-inflammatory cytokines such as interleukin 8 (IL-8) and TNFα [69,70,71]. The IFNs in turn induce expression of a large number of ISGs [52].

Type I IFNs have been described as key regulatory links between the innate and adaptive immune response due to their ability to activate genes that are involved in modulating such responses [72]. Of particular relevance to T1D is HLA-I, whose hyperexpression is a frequent observation in insulin-containing pancreatic islets of people with recent onset T1D [6,56,73]. HLA-I is an important ISG which has a central role in presenting cytosolic antigens to CD8+ T cells and has been implicated as one of the major determinants of the beta cell fate in autoimmune T1D [56,72].

A number of studies have implicated local IFN production within the islet milieu in driving autoimmunity and progression towards clinical T1D. Indeed, type I IFN has been detected directly within the islets of subjects with T1D [6], and a range of downstream ISGs, such as signal transducer and activator of transcription 1 (STAT1), IRF1, HLA-E and myxoma resistance protein 1 (Mx1, also known as MXA) are also elevated at both the RNA and protein level in T1D islets [74,75,76]. Furthermore, HLA-DQB1, an allele of HLA-II, is associated with increased risk of T1D [77,78], and analysis of whole-blood RNA samples from participants in the Finnish Type 1 Diabetes Prediction and Prevention (DIPP) study revealed that an innate immune signature is present in children carrying this allele before detection of islet autoimmunity (seroconversion) [79]. This signature remains active throughout the period to diagnosis of T1D [79]. Importantly, among the upregulated genes identified in the seroconverted children were genes belonging to the RIG-I signalling pathway as well as the transcription factors (TFs) IRF5 and IRF7, all of which are involved in antiviral innate immune responses. Increases in Eosinophil-derived neurotoxin (EDN) have also been seen in children progressing to clinical T1D [79], which is significant because EDN (also known as ribonuclease A family 2 (RNASE2)) is a ligand for TLR2 and has been shown to enhance dendritic cell (DC) maturation and migration, to promote helper T type 2 (Th2) immune responses and to propagate antiviral responses against ssRNA viruses [80]. Interestingly, EDN upregulation has also been reported in other autoimmune conditions, including systemic lupus erythematosus (SLE) and rheumatoid arthritis [81,82].

These provocative observations made from in vivo studies have received equally powerful support from in vitro studies in which isolated human islets were infected with CVB. Thus, a recent study showed that the infection of human islets with CVB3 induces the upregulation of type I and type III (IFNλ1 and IFNλ2) IFNs as well as a strong IFN signature manifest as increased ISGs such as ISG15, STAT1, IRF7, Mx1 and CXCL10 and PRRs MDA5, RIG-I and TLR3 [83].

It is also significant that a nonsynonymous SNP rs1990760 (A946T) found in *IFIH1*, the gene that encodes MDA5, is associated with T1D [84]. In particular, the TT and TC genotypes encoded by this SNP are associated with either an increased risk of, or protection from T1D, respectively. Hierarchical gene clustering of the TT and TC genotypes of SNP rs1990760 may also be associated with lower or higher relative ISG gene expression, respectively [83]. These data suggest that, whilst EVs can induce a strong IFN response in human islets, the precise outcome is influenced by variations in the gene sequence at such polymorphic sites. A study in a human hepatocyte cell line, Huh7, showed that the presence of the A946T polymorphism in *IFIH1* leads to production of a form of MDA5 capable of inducing a stronger IFNβ response than that seen with the more common variant. Notably, expression of the A946T variant MDA5 in *Ifih1*-depleted mouse embryonic fibroblasts (MEFs) resulted in a failure to mount an effective IFN response against virus infection [85]. Further investigation revealed that the A946T variant induces a conformational change which abrogates the ATPase activity of MDA5 [85] that is essential for filament disassembly and interaction with dsRNA [68]. These data strongly support the notion that this variant of MDA5 may fail to function effectively as a PRR but, paradoxically, it may still elicit tissue damage by producing a chronic IFN response even in the absence of a virus infection, as has been noted in SLE [86].

Variants of *IFIH1* have also been associated with type I interferonopathies (a group of monogenic innate immune disorders characterised by type I IFN overproduction) autoimmunity and autoinflammation [87]. On this basis, it has even been suggested that a localised islet interferonopathy may precede EV infection in genetically susceptible individuals and that the burden of a viral infection may then serve to exacerbate this pre-existing inflammatory condition [87]. As such, constitutive activation of MDA5 might perpetuate a vicious cycle in which IFNs and ISGs are induced inappropriately and lead ultimately to apoptosis and/or increased immunogenicity.

Tyrosine kinase 2 (TYK2) is another key regulator of type I and type III IFN signalling, and gene variants predicted to decrease TYK2 functionality are associated with a reduced risk of T1D and other autoimmune conditions [88]. One such variant is TYK2 SNP rs2304256 whose AA genotype has been associated with reduced IFNα-induced STAT1 phosphorylation [89]. Similarly, knockdown of TYK2 significantly reduces phosphorylation of STAT1 and STAT2, IFNα-induced HLA-I expression and poly I:C-induced apoptosis in EndoC-βH1 cells [89]. Consistent with these findings, a recent study showed that the inhibition of TYK2 using novel, selective, drugs prevents IFNα+IL-1β-induced apoptosis in human islets without affecting normal function and survival of CVB-infected beta cells or islets [90].

## 3. IFNs Induce Endoplasmic Reticulum Stress, Unfolded Protein Response and Apoptosis

In addition to HLA-I hyperexpression, islet cell ER stress (and the associated unfolded protein response (UPR)) have been described among the features of T1D [91]. For example, Marroqui et al. showed that IFNα induces HLA-I and markers of ER stress such as Binding Immunoglobulin Protein (BIP), C/EBP Homologous Protein (CHOP), Activating Transcription Factor 3 (ATF3) and spliced X-box Binding Protein 1 (XBP1s) in the human beta cell line EndoC-βH1. They also found similar responses in the islets of people with recent-onset T1D [91,92]. These changes were reversed in EndoC-βH1 cells following siRNA-mediated knockdown of *TYK2*, *STAT2* or *STAT1+STAT2* [91], indicating that TYK2 and STAT2 play an indispensable role in IFNα-induced HLA-I upregulation and ER stress. Similarly, knockdown of *STAT1* significantly reduced IFNγ-induced HLA-I expression. This is consistent with the known roles of these proteins as upstream factors involved in IFNα and IFNγ signalling.

IFNα can induce phosphorylation and subsequent formation of both STAT1/STAT2 hetero- and STAT2/STAT2 homodimers, whereby either dimer can, by recruiting IRF9, induce the expression of downstream genes by binding to IFN-stimulated response elements (ISRE). IFNγ signalling on the other hand is more restricted since it promotes the binding of phosphorylated STAT1/STAT1 homodimers to gamma interferon-activated sites (GAS) [93,94]. A compensatory overexpression of STAT2 following STAT1 knockdown (and vice versa) [91] may explain the redundancy of STAT1 for IFNα-mediated induction of HLA-I and ER stress markers.

ER stress is frequently characterised by a sustained activation of UPR—a mechanism which regulates the protein processing capacity of the ER during conditions such as virus infection [95] and which can culminate in apoptosis [96,97]. Three branches of the UPR have been described, each regulated by a principal ER transmembrane sensor, including dsRNA activated protein kinase (PKR)-like ER kinase (PERK), inositol requiring enzyme 1 (IRE1) and ATF6 [96]. The three branches work in parallel to sustain protein folding capacity, promote mRNA decay and reduce ER protein flux. Following activation of ATF6 and its subsequent cleavage in the Golgi apparatus, the cytosolic N-terminal region, ATF6(N) translocates to the nucleus to promote expression of ER resident chaperones, including BIP and glucose-regulated protein 94 (GRP94). Accumulation of unfolded proteins in the ER also induces oligomerisation and autophosphorylation of PERK. Activated PERK in turn phosphorylates eukaryotic Initiation Factor 2 subunit α (eIF2α), thereby inhibiting translation of the majority of cellular mRNAs. Exceptions are some mRNAs containing short open reading frames (ORFs) in their 5′ UTR: one such being ATF4. The renewed translation of ATF4 is enhanced under these conditions and this, in turn, promotes the expression of CHOP and ATF3. Notably, CHOP activation by this mechanism has been associated with apoptosis of heart and lung cells in mouse models of CVB3-induced acute myocarditis and pulmonary fibrosis [98,99]. Finally, the accumulation of unfolded proteins in the ER lumen promotes the activation and oligomerisation of IRE1α, a protein with dual kinase and endoribonuclease activity, which then targets XBP1 for splicing. The product (XBP1s) is a transcription factor which regulates various processes including lipid biosynthesis, expression of protein-folding chaperones and ER-associated protein degradation (ERAD) [100]. The RNase activity of IRE1α is also implicated in the mitigation of ER stress by enhancing mRNA degradation in a process known as IRE1α-dependent degradation (RIDD) [100].

IFNα-induced apoptosis in the B lymphoma cell line Daudi requires c-Jun N-terminal kinase 1 (JNK1) [101], and it may be significant that IFNγ + IL-1β also induce apoptosis and promote activation of IRE1α and JNK1 in EndoC-βH1 cells [92,102]. In addition, either IRE1α knockdown or the use of tauroursodeoxycholic acid (TUDCA), to attenuate ER stress, decreased JNK phosphorylation and cytokine-induced apoptosis in EndoC-βH1 cells [92]. Intriguingly, deletion of the *ire1α* gene results in a significant reduction in proinsulin synthesis, insulin content and insulin secretion without affecting preproinsulin mRNA levels in mouse beta cells. This correlated with increases in the ER stress markers CHOP, BIP and GRP94 [100]. IFNα-induced ER stress has also been shown to cause an increase in proinsulin to insulin ratio and to reduce beta cell insulin content as well as the expression of proinsulin convertase 1/3 (PC1/3) and PC2 in EndoC-βH1 and human islets. Pretreatment with TUDCA and 4-phenyl butyric acid (PBA) reversed all the IFNα-induced effects, suggesting that these changes are mediated by ER stress [103]. 

Several viruses (including hepatitis C and hepatitis B viruses, Japanese encephalitis virus and human cytomegalovirus) can activate both UPR and the IRE1α/XBP1 pathway to facilitate their own replication [104]. In addition, a recent study revealed that CVB5 infection triggers the PERK and IRE1α branches of UPR in EndoC-βH1 and in the rat beta cell line, INS-1E [105]. Following CVB5 infection, a fully activated IRE1α/XBP1s pathway promoted phosphorylation and activation of JNK1, leading to an increase in viral replication and, ultimately, apoptosis. Conversely, siRNA mediated *IRE1α* knockdown or chemical inhibition of JNK1 reduced both responses [105]. Importantly, the pro-apoptotic and virus-activating effects of JNK1 were regulated independently. These findings suggest that IRE1α-induced JNK1 activation may be required for efficient CVB replication in pancreatic beta cells. The same study also showed that despite activation of the PERK pathway in virally infected cells, none of the downstream ER stress markers—CHOP, ATF4 and ATF3—were induced, suggesting an incomplete activation of this branch of the UPR [105]. Enteroviruses depend on internal ribosome entry sites (IRES) to mediate protein translation and replication, and it has been shown that eIF2α is also required for IRES-dependent translation initiation [106]. These findings suggest that some EVs have evolved to take advantage of the selective UPR response occurring in beta cells during infection, but how they achieve this and what role IFNs play are still an open question.

## 4. IFNs May Promote the Expression of Neo-Antigens in Beta Cells

A recent study shed new light on the processes by which beta cell autoantigens may be generated during the progression to T1D and hinted that this process is exacerbated during islet inflammation and viral infection. In particular, it was noted that synthesis of insulin DRiPs (defective ribosomal products) may be increased under stress conditions [107]. Insulin DRiPs are proteins which arise from aberrant translation of the insulin mRNA by initiation at one or more AUG codons located downstream (3′) of the usual initiation site and which are out of frame with the insulin protein. Accordingly, the resultant protein has a unique sequence which bears no relation to the primary structure of insulin and which may, as a result, have heightened antigenicity. The authors demonstrated that the newly generated epitopes have a strong binding affinity for HLA-A2 and that CD8+ T cells directed against a DRiP epitope are present at significantly higher frequencies in the peripheral blood of people with T1D vs. healthy donors. Moreover, activated peptide-specific cytotoxic T cells (CTLs) can specifically kill beta cells obtained from HLA-A2+ T1D donors. Intriguingly, when islets were exposed to a combination of high glucose, IFNγ and IL-1β, the killing of beta cells was enhanced by activated INS-DRiP1-9-specific CTL, suggesting that inflammatory cytokines and ER stress may enhance production of INS-DRiP and T cell mediated beta cell killing [107].

Protein deamidation is a post-translational modification (PTM) which has been suggested as an underlying immunogenic mechanism for the generation of novel islet autoantigens in individuals with high risk HLA-DQ2/DQ8 [108]. Tissue transglutaminase (tTG) has deamidase activity and is expressed in human islets and DCs. Importantly, exposure to IFNγ + IL-1β enhanced tTG activity in islets and tolerogenic DC (tolDC). In addition, supernatants from activated GAD65-specific CD4+ T cells had elevated levels of inflammatory cytokines, including IFNγ, IL-1β and TNFα and exposure of islets to these supernatants promoted deamidation of two glutamine residues in the C-peptide region of proinsulin, whereas islets incubated with supernatants from resting CD4+ T cells produced only native C-peptide [108]. Furthermore, DCs from people heterozygous for HLA-DQ2/DQ8, but not those who are homozygous for HLA-DQ2 or HLA-DQ8, produced deamidated Islet Antigen 2 (IA-2) molecules when pulsed with native IA-2. Intriguingly, in comparison with native IA-2, the deamidated variants bind to DQ8trans and/or DQ8cis molecules with higher affinity than the native protein in cell-free assays [108]. To date, a number of T1D associated neo-antigens and the underlying PTM have been described [109,110,111,112,113,114,115,116]. These findings suggest that islet inflammation can potentially promote the formation of neo-antigens by post-translational modification of islet proteins, thereby creating an immunogenic milieu conducive to the development of autoimmunity.

## 5. Epigenetic Modulation of Cellular Responses by IFNs

Inflammatory cytokines can also modulate cellular responses at the epigenetic level. Changes in DNA methylation (DNAm) have been reported in whole blood and monocytes of T1D patients [117,118], and IFNs can significantly alter chromatin accessibility and DNAm patterns in islet cells [53,75,119]. A multi-omics analysis of human beta cells revealed extensive changes in chromatin accessibility associated with altered mRNA and protein expression following exposure to IFNα [75]. Intriguingly, whilst IFNα caused a marked increase in the proportion of accessible chromatin as early as 2 h after stimulation, these effects were significantly reduced after 24 h of IFNα exposure, indicative of a dual regulation involving ‘early’ and ‘late’ response regions [75]. The newly accessible open chromatin regions (OCRs) were enriched for TF binding motifs and mapped predominantly to regions downstream of transcription start sites (TSS). Importantly, the comparison of RNA-seq data revealed a significant overlap between the gene expression profiles seen in IFNα-treated islets (and beta cells) and those obtained from islets of people with T1D [120]. A similar correlation was not seen when islets from people with T2D were examined [75,121].

Epigenetic DNA hypomethylation is a feature of IFNα-treated islets, and the changes occur most often in pathways associated with the regulation of apoptosis, protein phosphorylation and fatty acid oxidation [53]. In addition, such alterations are over-represented in immune-related pathways, including those involved in antigen processing and presentation, and host defence responses to virus. Ingenuity Pathway Analysis (IPA) has pointed to STATs and IRFs as primary regulators of IFNα-induced transcripts, with IRF7 playing a dominant role. DNA demethylation is generally considered to facilitate gene transcription and integration of data relating methylation changes with mRNA upregulation during IFNα treatment identifies biological processes such as host defence responses to viral infection and innate immune responses [53]. The enzyme Ten-eleven translocation (TET) methylcytosine dioxygenase 2 (TET2) catalyses DNA demethylation by converting 5-methylcytosine (5mC) to 5-hydroxymethylcytosine (5hmC) and is activated in cells exposed to IFNα [53]. This correlates with an increase in the activity of polynucleotide phosphorylase (PNPase) also known as PNPT1, an exoribonuclease which degrades a specific miRNA, miR-26a, involved in the negative regulation of TET2 [53]. Hence, by inducing miR-26a degradation, IFNα indirectly promotes TET2-mediated DNA demethylation.

In addition to IFNα, other pro-inflammatory cytokines also induce changes in DNA methylation, histone modification and chromatin remodelling. Thus, newly accessible chromatin regions have also been identified in human islets and EndoC-βH1 cells exposed to IFNγ + IL-1β. In common with the OCRs induced by IFNα, induced regulatory elements (IREs) are characterised by the presence of abundant TF binding sites and are often localised distal to TSS. Further analysis identified two distinct IREs that become co-occupied and activated by inflammatory TFs upon cytokine exposure. Notably, these sites are enriched in polymorphic T1D risk variants [119].

Processes leading to miRNA dysregulation occur in cells during either acute or persistent infection with CVB. For example, 33 miRNAs were altered in expression by at least 3-fold when human islets were infected with CVB5 [122]. Similarly, in the pancreatic ductal cell line PANC-1, 81 miRNAs were altered during persistent infection with CVB4 [123]. In silico miRNA target scanning identified 57 and 49 candidate T1D risk genes as putative targets of the differentially expressed miRNAs in CVB5-infected islets and CVB4-infected PANC-1, respectively. Whilst these findings support the notion that miRNAs contribute to the altered cellular milieu during EV infection [124], both studies limited their search to target transcripts in T1D risk genes. Using a different approach, the expression of 57 miRNAs was altered by at least 2-fold in IFNγ + IL-1β-stimulated human islets [125]. Among these were miR-23b-3p and miR-149-5p which were also either down- (miR-23b-3p) or up-regulated (miR-149-5p) in CVB4-infected PANC-1 cells [123]. These changes led to alterations in various members of the Bcl-2 (B cell lymphoma 2) family, critical regulators of apoptosis, including P53 Up-regulated Modulator of Apoptosis (PUMA), Death Protein 5 (DP5), PMA-Induced Protein 1 (also known as NOXA), Bcl2 Associated X Protein (BAX) and Bcl-2-Like Protein 11 (also known as BIM) [125]. Thus, altered miRNA expression may be an important mechanism by which cells respond to IFNs and modulate sensitivity to apoptosis.

Finally, in addition to epigenetic processes, other aspects of islet cell gene expression are also modulated by IFNα. For example, exposure of EndoC-βH1 cells or human islets to IFNα influences the cellular splicing machinery leading to modified TSS usage and intron removal and generation of alternatively spliced proteins. This is important because such modifications may lead to intron retention which has often been associated with alterations in mRNA stability and translation efficiency [126]. Such modulatory effects of IFNα are in line with its antiviral role, since these effects are usually associated with the selective regulation of protein isoforms having either antiviral functions or roles in virus replication [75,127].

## 6. IFN-Mediated Antiviral Responses May Determine the Fate of Infected Cells

It has been suggested that defective immune responses against CVB infection could be linked to the loss of immune tolerance to insulin in children in their first years of life [128]. This is important because both type I and type III IFNs play a role in driving the host response to CVBs in human islets [83]. However, in order to offset this response, it has also been suggested that CVB3 may block production of type I and type III IFNs by islet cells [129]. Thus, Lind et al. noted that whereas treatment with the viral dsRNA mimetic, poly I:C, significantly increased the production of IFNλ1, IFNλ2 and IFNβ in HeLa cells, their expression remained unchanged after acute CVB3 infection [129].

Studies in individuals with Idiopathic Dilated Cardiomyopathy (IDCM) have revealed the development of 5′ terminal deletions (5′-TD) in the CVB3 RNA genome during viral persistence in cardiomyocytes [60]. This unexpected genome modification may then underlie the ability of the virus to persist in the cells over long periods. Consistent with this notion, detailed mechanistic studies imply that whereas 5′-TD CVB3 has an attenuated replication efficiency, these viruses can still maintain a low grade infection and support functionally competent processes that allow disruption of the host cell protein translation machinery [60]. It is not known whether 5′-TD accounts for the persistence of EVs following infection of human beta cells (although this has been proposed in mouse pancreas [130]), but the concept that persistent infection can be sustained in these cells is concordant with evidence of VP1 detection in a small number of beta cells of individuals with both recent onset and longer duration T1D [4,7,131]. In this scenario, it is proposed that an attenuated antiviral response may facilitate virus persistence, thereby allowing the host and virus to co-exist without the development of large-scale beta cell lysis. Studies of subjects with fulminant type 1 diabetes (which is also associated with EV infection) imply that the consequences of a full-blown lytic EV infection are catastrophic at the level of the endocrine pancreas [132]. It is, therefore, plausible that, in cases of T1D associated with sublytic EV infections, there is early production of type I and type III IFNs leading to upregulation of cellular ribonucleases (such as IFN-induced protein with tetratricopeptide repeats 1 (IFIT1), ribonuclease L (RNase L), 2′-5′-oligoadenylate synthetase 1 (OAS1), adenosine deaminase acting on RNA 1 (ADAR1) and PKR) [59] and that these promote the formation of 5′ deletions within the viral RNA to facilitate persistence.

## 7. IFNs as Regulators of Cell Mediated Immunity

It might be envisaged that continuous application of IFN-induced cellular stress would ultimately promote beta cell death and the generation of neo-antigens. Such neo-antigens might then be endocytosed and presented by plasmacytoid DCs (pDCs) to mediate the activation and maturation of autoreactive CD8+ T and CD4+ T cells [133]. Autoreactive CD8+ T and CD4+ T cells can in turn target the source of these autoantigens for specific killing (Figure 1). Activation, proliferation and differentiation of CD8+ T cells depends on the availability of three key factors including HLA-I-mediated antigen presentation, the presence of costimulatory signals and inflammatory cytokines [134,135]. Type I IFNs have been highlighted as important in this context [134].

PDCs are an important source of type I IFN, and their numbers are increased in individuals at risk of T1D as well as in those with recent onset and established T1D [136]. PDCs from people with T1D express higher levels of HLA-DR compared to those from healthy control subjects, and they produce IFNα when stimulated with CVB4. Exposure of CD4+ and CD8+ T cells from healthy donors to IFNα-producing pDCs enhanced the production of IFNγ, but this was even more evident in subjects with T1D [136]. These findings support a role for pDCs as a potential source of type I IFNs in T1D. A role for pDCs in the initiation of autoimmune diabetes has also been highlighted by observations that pDC depletion significantly delays the onset in non-obese diabetic (NOD) mice [137]. Importantly, a recent study has shown expression of HLA-II on a subset of pancreatic beta cells from donors with recent onset T1D [120]. Since HLA-II is normally only expressed by professional antigen presenting cells (APCs) such as DCs, it has been suggested that HLA-II-expressing beta cells may substitute for DCs by directly presenting islet autoantigens to CD4+ T cells [120]. It remains to be established whether the small subpopulation of HLA-II expressing beta cells is an important source of type I and type III IFNs, but this warrants consideration.

Programmed cell death protein 1 (PD-1) and its ligand PDL-1 play a key role in regulating the proliferation of Ag-specific CD8+ T cells, and they have become the focus of effective therapies targeting several malignancies [138]. In this context, it is noteworthy that increasing numbers of cases are being reported in which an unexpected outcome of such therapeutic strategies is the acute onset of a syndrome with the features of T1D [139]. This suggests that interference in PDL-1/PD-1 signalling may be detrimental to beta cell survival under certain circumstances. Consistent with this, we found that beta cell PDL-1 expression is induced in parallel with islet inflammation in human subjects with recent-onset T1D [76]. Thus, it seems possible that islets respond to inflammatory attack by upregulation of PDL-1 as a defensive mechanism to impede the actions of influent immune cells. Similarly, they also increase the expression of HLA-E under these conditions. HLA-E is a non-classical member of the HLA class I family, which also plays a role in islet protection by interaction with innate immune cells, such as NK cells, which may also be present in the infiltrates [75]. Importantly, both PDL-1 and HLA-E are induced by exposure of human islets to IFNα, and this may be secondary to induction of the transcription factor IRF1 [75,140].

Finally, in this context, we note that infection of pancreatic islets with CVB1-7 or stimulation of EndoC-βH1 cells with IL-1β + IFNγ promotes IL-32 expression [141]. IL-32 isoforms have been implicated in DC maturation and DC-induced chemotaxis of activated CD4+ and CD8+ T cells [142]. Interestingly, in HLA-DR-DQ conferred children who developed islet autoantibodies or T1D, elevated expression of IL-32 has been found in CD4+ and CD8+ T cells and NK cells [141].

## 8. Conclusions

Taken together, the studies summarised in this review imply that virally-induced production of type I and type III IFNs may be a critical factor that underpins the development of autoimmunity in genetically susceptible individuals who are progressing to type 1 diabetes. Interventions that minimise viral infection may, therefore, prove to be an effective means to slow the rate of progression in such individuals. As such, we await, with great interest and optimism, the outcome of clinical trials employing either antiviral agents or an anti-EV vaccine in relevant subjects.

## References

[1] Pugliese A. (2017). Autoreactive T cells in type 1 diabetes. J. Clin. Investig..

[2] Blanter M., Sork H., Tuomela S., Flodström-Tullberg M. (2019). Genetic and Environmental Interaction in Type 1 Diabetes: A Relationship Between Genetic Risk Alleles and Molecular Traits of Enterovirus Infection?. Curr. Diabetes Rep..

[3] Eizirik D., Pasquali L., Cnop M. (2020). Pancreatic β-cells in type 1 and type 2 diabetes mellitus: Different pathways to failure. Nat. Rev. Endocrinol..

[4] Richardson S.J., Willcox A., Bone A.J., Foulis A.K., Morgan N.G. (2009). The prevalence of enteroviral capsid protein vp1 immunostaining in pancreatic islets in human type 1 diabetes. Diabetologia.

[5] Anagandula M., Richardson S.J., Oberste M.S., Sioofy-Khojine A.B., Hyoty H., Morgan N.G., Korsgren O., Frisk G. (2014). Infection of human islets of Langerhans with two strains of Coxsackie B virus serotype 1: Assessment of virus replication, degree of cell death and induction of genes involved in the innate immunity pathway. J. Med. Virol..

[6] Foulis A.K., Farquharson M.A., Meager A. (1987). Immunoreactive alpha-interferon in insulin-secreting beta cells in type 1 diabetes mellitus. Lancet (Lond. Engl.).

[7] Krogvold L., Edwin B., Buanes T., Frisk G., Skog O., Anagandula M., Korsgren O., Undlien D., Eike M.C., Richardson S.J. (2015). Detection of a low-grade enteroviral infection in the islets of langerhans of living patients newly diagnosed with type 1 diabetes. Diabetes.

[8] Sioofy-Khojine A.B., Lehtonen J., Nurminen N., Laitinen O.H., Oikarinen S., Huhtala H., Pakkanen O., Ruokoranta T., Hankaniemi M.M., Toppari J. (2018). Coxsackievirus B1 infections are associated with the initiation of insulin-driven autoimmunity that progresses to type 1 diabetes. Diabetologia.

[9] Poma A., Genoni A., Broccolo F., Denaro M., Pugliese A., Basolo F., Toniolo A. (2020). Immune Transcriptome of Cells Infected with Enterovirus Strains Obtained from Cases of Type 1 Diabetes. Microorganisms.

[10] Richardson S., Morgan N. (2018). Enteroviral infections in the pathogenesis of type 1 diabetes: New insights for therapeutic intervention. Curr. Opin. Pharmacol..

[11] Elshebani A., Olsson A., Westman J., Tuvemo T., Korsgren O., Frisk G. (2007). Effects on isolated human pancreatic islet cells after infection with strains of enterovirus isolated at clinical presentation of type 1 diabetes. Virus Res..

[12] Hindersson M., Elshebani A., Orn A., Tuvemo T., Frisk G. (2005). Simultaneous type 1 diabetes onset in mother and son coincident with an enteroviral infection. J. Clin. Virol. Off. Publ. Pan Am. Soc. Clin. Virol..

[13] Nairn C., Galbraith D., Taylor K., Clements G. (1999). Enterovirus variants in the serum of children at the onset of Type 1 diabetes mellitus. Diabet. Med. J. Br. Diabet. Assoc..

[14] Moya-Suri V., Schlosser M., Zimmermann K., Rjasanowski I., Gürtler L., Mentel R. (2005). Enterovirus RNA sequences in sera of schoolchildren in the general population and their association with type 1-diabetes-associated autoantibodies. J. Med. Microbiol..

[15] Champsaur H., Dussaix E., Samolyk D., Fabre M., Bach C., Assan R. (1980). Diabetes and Coxsackie virus B5 infection. Lancet (Lond. Engl.).

[16] Díaz-Horta O., Bello M., Cabrera-Rode E., Suárez J., Más P., García I., Abalos I., Jofra R., Molina G., Díaz-Díaz O. (2001). Echovirus 4 and type 1 diabetes mellitus. Autoimmunity.

[17] Otonkoski T., Roivainen M., Vaarala O., Dinesen B., Leipälä J., Hovi T., Knip M. (2000). Neonatal Type I diabetes associated with maternal echovirus 6 infection: A case report. Diabetologia.

[18] Paananen A., Ylipaasto P., Rieder E., Hovi T., Galama J., Roivainen M. (2003). Molecular and biological analysis of echovirus 9 strain isolated from a diabetic child. J. Med. Virol..

[19] Smith C., Clements G., Riding M., Collins P., Bottazzo G., Taylor K. (1998). Simultaneous onset of type 1 diabetes mellitus in identical infant twins with enterovirus infection. Diabet. Med. A J. Br. Diabet. Assoc..

[20] Yeung W.C., Rawlinson W.D., Craig M.E. (2011). Enterovirus infection and type 1 diabetes mellitus: Systematic review and meta-analysis of observational molecular studies. BMJ (Clin. Res. Ed.).

[21] Stene L., Oikarinen S., Hyöty H., Barriga K., Norris J., Klingensmith G., Hutton J., Erlich H., Eisenbarth G., Rewers M. (2010). Enterovirus infection and progression from islet autoimmunity to type 1 diabetes: The Diabetes and Autoimmunity Study in the Young (DAISY). Diabetes.

[22] Werf N.v.d., Kroese F.G.M., Rozing J., Hillebrands J.L. (2007). Viral Infections as Potential Triggers of Type 1 Diabetes. Diabetes/Metab. Res. Rev..

[23] Craig M., Nair S., Stein H., Rawlinson W. (2013). Viruses and type 1 diabetes: A new look at an old story. Pediatric Diabetes.

[24] Coppieters K., Boettler T., Herrath M.v. (2012). Virus infections in type 1 diabetes. Cold Spring Harb. Perspect. Med..

[25] Christen U., Herrath M.v. (2011). Do viral infections protect from or enhance type 1 diabetes and how can we tell the difference?. Cell. Mol. Immunol..

[26] Tracy S., Drescher K., Jackson J., Kim K., Kono K. (2010). Enteroviruses, type 1 diabetes and hygiene: A complex relationship. Rev. Med. Virol..

[27] Ifie E., Russell M.A., Dhayal S., Leete P., Sebastiani G., Nigi L., Dotta F., Marjomaki V., Eizirik D.L., Morgan N.G. (2018). Unexpected subcellular distribution of a specific isoform of the Coxsackie and adenovirus receptor, CAR-SIV, in human pancreatic beta cells. Diabetologia.

[28] Oikarinen M., Tauriainen S., Honkanen T., Vuori K., Karhunen P., Vasama-Nolvi C., Oikarinen S., Verbeke C., Blair G.E., Rantala I. (2008). Analysis of pancreas tissue in a child positive for islet cell antibodies. Diabetologia.

[29] Schulte B., Bakkers J., Lanke K., Melchers W., Westerlaken C., Allebes W., Aanstoot H., Bruining G., Adema G., Kuppeveld F.V. (2010). Detection of enterovirus RNA in peripheral blood mononuclear cells of type 1 diabetic patients beyond the stage of acute infection. Viral Immunol..

[30] Ylipaasto P., Klingel K., Lindberg A.M., Otonkoski T., Kandolf R., Hovi T., Roivainen M. (2004). Enterovirus infection in human pancreatic islet cells, islet tropism in vivo and receptor involvement in cultured islet beta cells. Diabetologia.

[31] Hiltunen M., Hyöty H., Knip M., Ilonen J., Reijonen H., Vähäsalo P., Roivainen M., Lönnrot M., Leinikki P., Hovi T. (1997). Islet Cell Antibody Seroconversion in Children Is Temporally Associated with Enterovirus Infections. J. Infect. Dis..

[32] Salvatoni A., Baj A., Bianchi G., Federico G., Colombo M., Toniolo A. (2013). Intrafamilial spread of enterovirus infections at the clinical onset of type 1 diabetes. Pediatr. Diabetes.

[33] Lönnrot M., Salminen K., Knip M., Savola K., Kulmala P., Leinikki P., Hyypiä T., Akerblom H., Hyöty H. (2000). Enterovirus RNA in serum is a risk factor for beta-cell autoimmunity and clinical type 1 diabetes: A prospective study. Childhood Diabetes in Finland (DiMe) Study Group. J. Med. Virol..

[34] Wagenknecht L., Roseman J., Herman W. (1991). Increased incidence of insulin-dependent diabetes mellitus following an epidemic of Coxsackievirus B5. Am. J. Epidemiol..

[35] Gamble D., Taylor K. (1969). Seasonal incidence of diabetes mellitus. Br. Med. J..

[36] Laitinen O.H., Honkanen H., Pakkanen O., Oikarinen S., Hankaniemi M.M., Huhtala H., Ruokoranta T., Lecouturier V., Andre P., Harju R. (2014). Coxsackievirus B1 is associated with induction of beta-cell autoimmunity that portends type 1 diabetes. Diabetes.

[37] Oikarinen S., Tauriainen S., Hober D., Lucas B., Vazeou A., Sioofy-Khojine A., Bozas E., Muir P., Honkanen H., Ilonen J. (2014). Virus antibody survey in different European populations indicates risk association between coxsackievirus B1 and type 1 diabetes. Diabetes.

[38] Vehik K., Lynch K.F., Wong M.C., Tian X., Ross M.C., Gibbs R.A., Ajami N.J., Petrosino J.F., Rewers M., Toppari J. (2019). Prospective virome analyses in young children at increased genetic risk for type 1 diabetes. Nat. Med..

[39] Rodriguez-Calvo T., Sabouri S., Anquetil F., Herrath M.v. (2016). The Viral Paradigm in Type 1 Diabetes: Who Are the Main Suspects?. Autoimmun. Rev..

[40] Rogers M., Basu T., Kim C. (2019). Lower Incidence Rate of Type 1 Diabetes after Receipt of the Rotavirus Vaccine in the United States, 2001–2017. Sci. Rep..

[41] Burke R., Tate J., Jiang B., Parashar U. (2020). Rotavirus and Type 1 Diabetes-is there a connection? A synthesis of the evidence. J. Infect. Dis..

[42] Larsson P., Lakshmikanth T., Laitinen O., Utorova R., Jacobson S., Oikarinen M., Domsgen E., Koivunen M., Chaux P., Devard N. (2015). A preclinical study on the efficacy and safety of a new vaccine against Coxsackievirus B1 reveals no risk for accelerated diabetes development in mouse models. Diabetologia.

[43] Stone V.M., Hankaniemi M.M., Svedin E., Sioofy-Khojine A., Oikarinen S., Hyoty H., Laitinen O.H., Hytonen V.P., Flodstrom-Tullberg M. (2018). A Coxsackievirus B vaccine protects against virus-induced diabetes in an experimental mouse model of type 1 diabetes. Diabetologia.

[44] Stone V., Hankaniemi M., Laitinen O., Sioofy-Khojine A., Lin A., Lozano I.D., Mazur M., Marjomäki V., Loré K., Hyöty H. (2020). A hexavalent Coxsackievirus B vaccine is highly immunogenic and has a strong protective capacity in mice and nonhuman primates. Sci. Adv..

[45] Zaccone P., Fehervari Z., Phillips J., Dunne D., Cooke A. (2006). Parasitic worms and inflammatory diseases. Parasite Immunol..

[46] Bodansky H., Staines A., Stephenson C., Haigh D., Cartwright R. (1992). Evidence for an environmental effect in the aetiology of insulin dependent diabetes in a transmigratory population. BMJ (Clin. Res. Ed.).

[47] Oilinki T., Otonkoski T., Ilonen J., Knip M., Miettinen P. (2012). Prevalence and characteristics of diabetes among Somali children and adolescents living in Helsinki, Finland. Pediatric Diabetes.

[48] Söderström U., Aman J., Hjern A. (2012). Being born in Sweden increases the risk for type 1 diabetes - a study of migration of children to Sweden as a natural experiment. Acta Paediatr. (Oslo, Nor. 1992).

[49] Viskari H., Ludvigsson J., Uibo R., Salur L., Marciulionyte D., Hermann R., Soltesz G., Füchtenbusch M., Ziegler A., Kondrashova A. (2005). Relationship between the incidence of type 1 diabetes and maternal enterovirus antibodies: Time trends and geographical variation. Diabetologia.

[50] Viskari H., Ludvigsson J., Uibo R., Salur L., Marciulionyte D., Hermann R., Soltesz G., Füchtenbusch M., Ziegler A., Kondrashova A. (2004). Relationship between the incidence of type 1 diabetes and enterovirus infections in different European populations: Results from the EPIVIR project. J. Med. Virol..

[51] Seiskari T., Kondrashova A., Viskari H., Kaila M., Haapala A., Aittoniemi J., Virta M., Hurme M., Uibo R., Knip M. (2007). Allergic sensitization and microbial load--a comparison between Finland and Russian Karelia. Clin. Exp. Immunol..

[52] Mostafavi S., Yoshida H., Moodley D., LeBoité H., Rothamel K., Raj T., Ye C., Chevrier N., Zhang S., Feng T. (2016). Parsing the Interferon Transcriptional Network and Its Disease Associations. Cell.

[53] Stefan-Lifshitz M., Karakose E., Cui L., Ettela A., Yi Z., Zhang W., Tomer Y. (2019). Epigenetic Modulation of β Cells by Interferon-α via PNPT1/mir-26a/TET2 Triggers Autoimmune Diabetes. JCI Insight.

[54] Lind K., Richardson S.J., Leete P., Morgan N.G., Korsgren O., Flodstrom-Tullberg M. (2013). Induction of an antiviral state and attenuated coxsackievirus replication in type III interferon-treated primary human pancreatic islets. J. Virol..

[55] Willcox A., Richardson S.J., Bone A.J., Foulis A.K., Morgan N.G. (2009). Analysis of islet inflammation in human type 1 diabetes. Clin. Exp. Immunol..

[56] Richardson S.J., Rodriguez-Calvo T., Gerling I.C., Mathews C.E., Kaddis J.S., Russell M.A., Zeissler M., Leete P., Krogvold L., Dahl-Jorgensen K. (2016). Islet cell hyperexpression of HLA class I antigens: A defining feature in type 1 diabetes. Diabetologia.

[57] Junior A.D., Sampaio N., Rehwinkel J. (2019). A Balancing Act: MDA5 in Antiviral Immunity and Autoinflammation. Trends Microbiol..

[58] Ivashkiv L.B., Donlin L.T. (2014). Regulation of Type I Interferon Responses. Nat. Rev. Immunol..

[59] Schlee M., Hartmann G. (2016). Discriminating Self From Non-Self in Nucleic Acid Sensing. Nat. Rev. Immunol..

[60] Bouin A., Gretteau P., Wehbe M., Renois F., N’Guyen Y., Lévêque N., Vu M., Tracy S., Chapman N., Bruneval P. (2019). Enterovirus Persistence in Cardiac Cells of Patients With Idiopathic Dilated Cardiomyopathy Is Linked to 5’ Terminal Genomic RNA-Deleted Viral Populations With Viral-Encoded Proteinase Activities. Circulation.

[61] Tam P., Messner R. (1999). Molecular Mechanisms of Coxsackievirus Persistence in Chronic Inflammatory Myopathy: Viral RNA Persists Through Formation of a Double-Stranded Complex Without Associated Genomic Mutations or Evolution. J. Virol..

[62] Chia J. (2005). The Role of Enterovirus in Chronic Fatigue Syndrome. J. Clin. Pathol..

[63] Jensen S., Thomsen A. (2012). Sensing of RNA Viruses: A Review of Innate Immune Receptors Involved in Recognizing RNA Virus Invasion. J. Virol..

[64] Pichlmair A., Sousa C.R.e. (2007). Innate Recognition of Viruses. Immunity.

[65] Geravandi S., Liu H., Maedler K. (2020). Enteroviruses and T1D: Is It the Virus, the Genes or Both which Cause T1D. Microorganisms.

[66] Pichlmair A., Schulz O., Tan C., Näslund T., Liljeström P., Weber F., Sousa C.R.e. (2006). RIG-I-mediated Antiviral Responses to Single-Stranded RNA Bearing 5’-phosphates. Science.

[67] Pichlmair A., Schulz O., Tan C.P., Rehwinkel J., Kato H., Takeuchi O., Akira S., Way M., Schiavo G., Reis e Sousa C. (2009). Activation of MDA5 Requires Higher-Order RNA Structures Generated during Virus Infection. J. Virol..

[68] Peisley A., Lin C., Wu B., Orme-Johnson M., Liu M., Walz T., Hur S. (2011). Cooperative Assembly and Dynamic Disassembly of MDA5 Filaments for Viral dsRNA Recognition. Proc. Natl. Acad. Sci. USA.

[69] Zhong B., Yang Y., Li S., Wang Y., Li Y., Diao F., Lei C., He X., Zhang L., Tien P. (2008). The Adaptor Protein MITA Links Virus-Sensing Receptors to IRF3 Transcription Factor Activation. Immunity.

[70] Guo B., Cheng G. (2007). Modulation of the Interferon Antiviral Response by the TBK1/IKKi Adaptor Protein TANK. J. Biol. Chem..

[71] Jacobs J., Coyne C. (2013). Mechanisms of MAVS Regulation at the Mitochondrial Membrane. J. Mol. Biol..

[72] Newby B., Mathews C. (2017). Type I Interferon Is a Catastrophic Feature of the Diabetic Islet Microenvironment. Front. Endocrinol..

[73] Foulis A.K., Farquharson M.A., Hardman R. (1987). Aberrant expression of class II major histocompatibility complex molecules by B cells and hyperexpression of class I major histocompatibility complex molecules by insulin containing islets in type 1 (insulin-dependent) diabetes mellitus. Diabetologia.

[74] Lundberg M., Krogvold L., Kuric E., Dahl-Jørgensen K., Skog O. (2016). Expression of Interferon-Stimulated Genes in Insulitic Pancreatic Islets of Patients Recently Diagnosed With Type 1 Diabetes. Diabetes.

[75] Colli M., Ramos-Rodríguez M., Nakayasu E., Alvelos M., Lopes M., Hill J., Turatsinze J., Brachène A.C.d., Russell M., Raurell-Vila H. (2020). An Integrated Multi-Omics Approach Identifies the Landscape of Interferon-α-Mediated Responses of Human Pancreatic Beta Cells. Nat. Commun..

[76] Colli M., Hill J., Marroquí L., Chaffey J., Santos R.D., Leete P., Brachène A.C.d., Paula F., Beeck A.O.d., Castela A. (2018). PDL1 Is Expressed in the Islets of People With Type 1 Diabetes and Is Up-Regulated by interferons-α and-γ via IRF1 Induction. EBioMedicine.

[77] Zhang M., Lin S., Yuan X., Lin Z., Huang Z. (2019). HLA-DQB1 and HLA-DRB1 Variants Confer Susceptibility to Latent Autoimmune Diabetes in Adults: Relative Predispositional Effects Among Allele Groups. Genes.

[78] Farina F., Picascia S., Pisapia L., Barba P., Vitale S., Franzese A., Mozzillo E., Gianfrani C., Pozzo G.G.D. (2019). HLA-DQA1 and HLA-DQB1 Alleles, Conferring Susceptibility to Celiac Disease and Type 1 Diabetes, Are More Expressed Than Non-Predisposing Alleles and Are Coordinately Regulated. Cells.

[79] Kallionpää H., Elo L., Laajala E., Mykkänen J., Ricaño-Ponce I., Vaarma M., Laajala T., Hyöty H., Ilonen J., Veijola R. (2014). Innate Immune Activity Is Detected Prior to Seroconversion in Children With HLA-conferred Type 1 Diabetes Susceptibility. Diabetes.

[80] Yang D., Chen Q., Su S., Zhang P., Kurosaka K., Caspi R., Michalek S., Rosenberg H., Zhang N., Oppenheim J. (2008). Eosinophil-derived Neurotoxin Acts as an Alarmin to Activate the TLR2-MyD88 Signal Pathway in Dendritic Cells and Enhances Th2 Immune Responses. J. Exp. Med..

[81] Bennett L., Palucka A., Arce E., Cantrell V., Borvak J., Banchereau J., Pascual V. (2003). Interferon and Granulopoiesis Signatures in Systemic Lupus Erythematosus Blood. J. Exp. Med..

[82] Bovin L., Rieneck K., Workman C., Nielsen H., Sørensen S., Skjødt H., Florescu A., Brunak S., Bendtzen K. (2004). Blood Cell Gene Expression Profiling in Rheumatoid Arthritis. Discriminative Genes and Effect of Rheumatoid Factor. Immunol. Lett..

[83] Domsgen E., Lind K., Kong L., Huhn M.H., Rasool O., van Kuppeveld F., Korsgren O., Lahesmaa R., Flodstrom-Tullberg M. (2016). An IFIH1 gene polymorphism associated with risk for autoimmunity regulates canonical antiviral defence pathways in Coxsackievirus infected human pancreatic islets. Sci. Rep..

[84] Oram R.A., Patel K., Hill A., Shields B., McDonald T.J., Jones A., Hattersley A.T., Weedon M.N. (2016). A Type 1 Diabetes Genetic Risk Score Can Aid Discrimination Between Type 1 and Type 2 Diabetes in Young Adults. Diabetes Care.

[85] Funabiki M., Kato H., Miyachi Y., Toki H., Motegi H., Inoue M., Minowa O., Yoshida A., Deguchi K., Sato H. (2014). Autoimmune Disorders Associated With Gain of Function of the Intracellular Sensor MDA5. Immunity.

[86] Gateva V., Sandling J., Hom G., Taylor K., Chung S., Sun X., Ortmann W., Kosoy R., Ferreira R., Nordmark G. (2009). A Large-Scale Replication Study Identifies TNIP1, PRDM1, JAZF1, UHRF1BP1 and IL10 as Risk Loci for Systemic Lupus Erythematosus. Nat. Genet..

[87] Jean-Baptiste V., Xia C., Clare-Salzler M., Horwitz M. (2017). Type 1 Diabetes and Type 1 Interferonopathies: Localization of a Type 1 Common Thread of Virus Infection in the Pancreas. EBioMedicine.

[88] Tao J., Zou Y., Feng X., Li J., Wang F., Pan F., Ye D. (2011). Meta-analysis of TYK2 Gene Polymorphisms Association With Susceptibility to Autoimmune and Inflammatory Diseases. Mol. Biol. Rep..

[89] Marroqui L., Santos R.D., Fløyel T., Grieco F., Santin I., Beeck A.O.d., Marselli L., Marchetti P., Pociot F., Eizirik D. (2015). TYK2, a Candidate Gene for Type 1 Diabetes, Modulates Apoptosis and the Innate Immune Response in Human Pancreatic β-Cells. Diabetes.

[90] Brachène A.C.d., Castela A., Beeck A.O.d., Mirmira R., Marselli L., Marchetti P., Masse C., Miao W., Leit S., Evans-Molina C. (2020). Pre-clinical Evaluation of TYK2 Inhibitors for Human Beta Cell Protection in Type 1 Diabetes. Diabetes Obes. Metab..

[91] Marroqui L., Santos R.D., Beeck A.O.d., Brachène A.C.d., Marselli L., Marchett P., Eizirik D. (2017). Interferon-α Mediates Human Beta Cell HLA Class I Overexpression, Endoplasmic Reticulum Stress and Apoptosis, Three Hallmarks of Early Human Type 1 Diabetes. Diabetologia.

[92] Brozzi F., Nardelli T.R., Lopes M., Millard I., Barthson J., Igoillo-Esteve M., Grieco F.A., Villate O., Oliveira J.M., Casimir M. (2015). Cytokines induce endoplasmic reticulum stress in human, rat and mouse beta cells via different mechanisms. Diabetologia.

[93] Majoros A., Platanitis E., Kernbauer-Hölzl E., Rosebrock F., Müller M., Decker T. (2017). Canonical and Non-Canonical Aspects of JAK-STAT Signaling: Lessons From Interferons for Cytokine Responses. Front. Immunol..

[94] Abdul-Sater A.A., Majoros A., Plumlee C.R., Perry S., Gu A.D., Lee C., Shresta S., Decker T., Schindler C. (2015). Different STAT Transcription Complexes Drive Early and Delayed Responses to Type I IFNs. J. Immunol. (Baltimore Md. 1950).

[95] Fung T.S., Torres J., Liu D.X. (2015). The Emerging Roles of Viroporins in ER Stress Response and Autophagy Induction during Virus Infection. Viruses.

[96] Walter P., Ron D. (2011). The Unfolded Protein Response: From Stress Pathway to Homeostatic Regulation. Science.

[97] Hu H., Tian M., Ding C., Yu S. (2019). The C/EBP Homologous Protein (CHOP) Transcription Factor Functions in Endoplasmic Reticulum Stress-Induced Apoptosis and Microbial Infection. Front. Immunol..

[98] Cai Z., Shen L., Ma H., Yang J., Yang D., Chen H., Wei J., Lu Q., Wang D., Xiang W. (2015). Involvement of Endoplasmic Reticulum Stress-Mediated C/EBP Homologous Protein Activation in Coxsackievirus B3-Induced Acute Viral Myocarditis. Circ. Heart Fail..

[99] Delbrel E., Soumare A., Naguez A., Label R., Bernard O., Bruhat A., Fafournoux P., Tremblais G., Marchant D., Gille T. (2018). HIF-1α Triggers ER Stress and CHOP-mediated Apoptosis in Alveolar Epithelial Cells, a Key Event in Pulmonary Fibrosis. Sci. Rep..

[100] Hassler J., Scheuner D., Wang S., Han J., Kodali V., Li P., Nguyen J., George J., Davis C., Wu S. (2015). The IRE1α/XBP1s Pathway Is Essential for the Glucose Response and Protection of β Cells. PLoS Biol..

[101] Yanase N., Hata K., Shimo K., Hayashida M., Evers B., Mizuguchi J. (2005). Requirement of c-Jun NH2-terminal Kinase Activation in Interferon-Alpha-Induced Apoptosis Through Upregulation of Tumor Necrosis Factor-Related Apoptosis-Inducing Ligand (TRAIL) in Daudi B Lymphoma Cells. Exp. Cell Res..

[102] Brozzi F., Gerlo S., Grieco F., Juusola M., Balhuizen A., Lievens S., Gysemans C., Bugliani M., Mathieu C., Marchetti P. (2016). Ubiquitin D Regulates IRE1α/c-Jun N-terminal Kinase (JNK) Protein-dependent Apoptosis in Pancreatic Beta Cells. J. Biol. Chem..

[103] Lombardi A., Tomer Y. (2017). Interferon Alpha Impairs Insulin Production in Human Beta Cells via Endoplasmic Reticulum Stress. J. Autoimmun..

[104] Li B., Gao B., Ye L., Han X., Wang W., Kong L., Fang X., Zeng Y., Zheng H., Li S. (2007). Hepatitis B Virus X Protein (HBx) Activates ATF6 and IRE1-XBP1 Pathways of Unfolded Protein Response. Virus Res..

[105] Colli M.L., Paula F.M., Marselli L., Marchetti P., Roivainen M., Eizirik D.L., Op de Beeck A. (2019). Coxsackievirus B Tailors the Unfolded Protein Response to Favour Viral Amplification in Pancreatic beta Cells. J. Innate Immun..

[106] Gerlitz G., Jagus R., Elroy-Stein O. (2002). Phosphorylation of Initiation factor-2 Alpha Is Required for Activation of Internal Translation Initiation During Cell Differentiation. Eur. J. Biochem..

[107] Kracht M., Lummel M.v., Nikolic T., Joosten A., Laban S., Slik A.v.d., Veelen P.v., Carlotti F., Koning E.d., Hoeben R. (2017). Autoimmunity Against a Defective Ribosomal Insulin Gene Product in Type 1 Diabetes. Nat. Med..

[108] McLaughlin R., Haan A.D., Zaldumbide A., Koning E.D., Ru A.D., Veelen P.V., Lummel M.V., Roep B. (2016). Human Islets and Dendritic Cells Generate Post-Translationally Modified Islet Autoantigens. Clin. Exp. Immunol..

[109] Marré M., James E., Piganelli J. (2015). β Cell ER Stress and the Implications for Immunogenicity in Type 1 Diabetes. Front. Cell Dev. Biol..

[110] Babon J.A., DeNicola M.E., Blodgett D.M., Crevecoeur I., Buttrick T.S., Maehr R., Bottino R., Naji A., Kaddis J., Elyaman W. (2016). Analysis of self-antigen specificity of islet-infiltrating T cells from human donors with type 1 diabetes. Nat. Med..

[111] Baker R., Rihanek M., Hohenstein A., Nakayama M., Michels A., Gottlieb P., Haskins K., Delong T. (2019). Hybrid Insulin Peptides Are Autoantigens in Type 1 Diabetes. Diabetes.

[112] Arribas-Layton D., Guyer P., Delong T., Dang M., Chow I., Speake C., Greenbaum C., Kwok W., Baker R., Haskins K. (2020). Hybrid Insulin Peptides Are Recognized by Human T Cells in the Context of DRB1*04:01. Diabetes.

[113] Wiles T., Powell R., Michel R., Beard K., Hohenstein A., Bradley B., Reisdorph N., Haskins K., Delong T. (2019). Identification of Hybrid Insulin Peptides (HIPs) in Mouse and Human Islets by Mass Spectrometry. J. Proteome Res..

[114] Delong T., Wiles T., Baker R., Bradley B., Barbour G., Reisdorph R., Armstrong M., Powell R., Reisdorph N., Kumar N. (2016). Pathogenic CD4 T Cells in Type 1 Diabetes Recognize Epitopes Formed by Peptide Fusion. Science.

[115] Marre M., McGinty J., Chow I., DeNicola M., Beck N., Kent S., Powers A., Bottino R., Harlan D., Greenbaum C. (2018). Modifying Enzymes Are Elicited by ER Stress, Generating Epitopes That Are Selectively Recognized by CD4+ T Cells in Patients With Type 1 Diabetes. Diabetes.

[116] Gonzalez-Duque S., Azoury M.E., Colli M.L., Afonso G., Turatsinze J.V., Nigi L., Lalanne A.I., Sebastiani G., Carre A., Pinto S. (2018). Conventional and Neo-antigenic Peptides Presented by beta Cells Are Targeted by Circulating Naive CD8+ T Cells in Type 1 Diabetic and Healthy Donors. Cell Metab..

[117] Fradin D., Fur S.L., Mille C., Naoui N., Groves C., Zelenika D., McCarthy M., Lathrop M., Bougnères P. (2012). Association of the CpG Methylation Pattern of the Proximal Insulin Gene Promoter With Type 1 Diabetes. PLoS ONE.

[118] Chen Z., Miao F., Paterson A.D., Lachin J.M., Zhang L., Schones D.E., Wu X., Wang J., Tompkins J.D., Genuth S. (2016). Epigenomic Profiling Reveals an Association Between Persistence of DNA Methylation and Metabolic Memory in the DCCT/EDIC Type 1 Diabetes Cohort. Proc. Natl. Acad. Sci. USA.

[119] Ramos-Rodríguez M., Raurell-Vila H., Colli M., Alvelos M., Subirana-Granés M., Juan-Mateu J., Norris R., Turatsinze J., Nakayasu E., Webb-Robertson B. (2019). The Impact of Proinflammatory Cytokines on the β-cell Regulatory Landscape Provides Insights Into the Genetics of Type 1 Diabetes. Nat. Genet..

[120] Russell M., Redick S., Blodgett D., Richardson S., Leete P., Krogvold L., Dahl-Jørgensen K., Bottino R., Brissova M., Spaeth J. (2019). HLA Class II Antigen Processing and Presentation Pathway Components Demonstrated by Transcriptome and Protein Analyses of Islet β-Cells From Donors With Type 1 Diabetes. Diabetes.

[121] Xin Y., Kim J., Okamoto H., Ni M., Wei Y., Adler C., Murphy A., Yancopoulos G., Lin C., Gromada J. (2016). RNA Sequencing of Single Human Islet Cells Reveals Type 2 Diabetes Genes. Cell Metab..

[122] Kim K.W., Ho A., Alshabee-Akil A., Hardikar A.A., Kay T.W., Rawlinson W.D., Craig M.E. (2016). Coxsackievirus B5 Infection Induces Dysregulation of microRNAs Predicted to Target Known Type 1 Diabetes Risk Genes in Human Pancreatic Islets. Diabetes.

[123] Engelmann I., Alidjinou E., Bertin A., Bossu J., Villenet C., Figeac M., Sane F., Hober D. (2017). Persistent Coxsackievirus B4 Infection Induces microRNA Dysregulation in Human Pancreatic Cells. Cell. Mol. Life Sci..

[124] Engelmann I., Alidjinou E., Bertin A., Sane F., Hober D. (2018). miRNAs in Enterovirus Infection. Crit. Rev. Microbiol..

[125] Grieco F., Sebastiani G., Juan-Mateu J., Villate O., Marroqui L., Ladrière L., Tugay K., Regazzi R., Bugliani M., Marchetti P. (2017). MicroRNAs miR-23a-3p, miR-23b-3p, and miR-149-5p Regulate the Expression of Proapoptotic BH3-Only Proteins DP5 and PUMA in Human Pancreatic β-Cells. Diabetes.

[126] Jacob A., Smith C. (2017). Intron Retention as a Component of Regulated Gene Expression Programs. Hum. Genet..

[127] Meertens L., Hafirassou M., Couderc T., Bonnet-Madin L., Kril V., Kümmerer B., Labeau A., Brugier A., Simon-Loriere E., Burlaud-Gaillard J. (2019). FHL1 Is a Major Host Factor for Chikungunya Virus Infection. Nature.

[128] Ashton M., Eugster A., Walther D., Daehling N., Riethausen S., Kuehn D., Klingel K., Beyerlein A., Zillmer S., Ziegler A. (2016). Incomplete Immune Response to Coxsackie B Viruses Associates With Early Autoimmunity Against Insulin. Sci. Rep..

[129] Lind K., Svedin E., Domsgen E., Kapell S., Laitinen O.H., Moll M., Flodstrom-Tullberg M. (2016). Coxsackievirus counters the host innate immune response by blocking type III interferon expression. J. Gen. Virol..

[130] Tracy S., Smithee S., Alhazmi A., Chapman N. (2015). Coxsackievirus can persist in murine pancreas by deletion of 5’ terminal genomic sequences. J. Med. Virol..

[131] Richardson S.J., Leete P., Bone A.J., Foulis A.K., Morgan N.G. (2013). Expression of the enteroviral capsid protein VP1 in the islet cells of patients with type 1 diabetes is associated with induction of protein kinase R and downregulation of Mcl-1. Diabetologia.

[132] Morgan N.G., Richardson S.J. (2014). Enteroviruses as causative agents in type 1 diabetes: Loose ends or lost cause?. Trends Endocrinol. Metab. Tem.

[133] Swiecki M., Colonna M. (2015). The Multifaceted Biology of Plasmacytoid Dendritic Cells. Nat. Rev. Immunol..

[134] Crouse J., Kalinke U., Oxenius A. (2015). Regulation of antiviral T cell responses by type I interferons. Nat. Rev. Immunol..

[135] Crouse J., Bedenikovic G., Wiesel M., Ibberson M., Xenarios I., Laer D.V., Kalinke U., Vivier E., Jonjic S., Oxenius A. (2014). Type I Interferons Protect T Cells Against NK Cell Attack Mediated by the Activating Receptor NCR1. Immunity.

[136] Xia C., Peng R., Chernatynskaya A., Yuan L., Carter C., Valentine J., Sobel E., Atkinson M., Clare-Salzler M. (2014). Increased IFN-α-producing Plasmacytoid Dendritic Cells (pDCs) in Human Th1-mediated Type 1 Diabetes: pDCs Augment Th1 Responses Through IFN-α Production. J. Immunol. (Baltimore Md. 1950).

[137] Diana J., Simoni Y., Furio L., Beaudoin L., Agerberth B., Barrat F., Lehuen A. (2013). Crosstalk between neutrophils, B-1a cells and plasmacytoid dendritic cells initiates autoimmune diabetes. Nat. Med..

[138] André P., Denis C., Soulas C., Bourbon-Caillet C., Lopez J., Arnoux T., Bléry M., Bonnafous C., Gauthier L., Morel A. (2018). Anti-NKG2A mAb Is a Checkpoint Inhibitor That Promotes Anti-tumor Immunity by Unleashing Both T and NK Cells. Cell.

[139] Ntali G., Kassi E., Alevizaki M. (2017). Endocrine sequelae of immune checkpoint inhibitors. Horm. (Athens Greece).

[140] Nekoua M.P., Dechaumes A., Sane F., Alidjinou E.K., Moutairou K., Yessoufou A., Hober D. (2020). Enteroviral Pathogenesis of Type 1 Diabetes: The Role of Natural Killer Cells. Microorganisms.

[141] Kallionpää H., Somani J., Tuomela S., Ullah U., Albuquerque R.d., Lönnberg T., Komsi E., Siljander H., Honkanen J., Härkönen T. (2019). Early Detection of Peripheral Blood Cell Signature in Children Developing β-Cell Autoimmunity at a Young Age. Diabetes.

[142] Son M., Jung M., Choi S., Cho D., Kim T. (2014). IL-32γ Induces Chemotaxis of Activated T Cells via Dendritic Cell-Derived CCL5. Biochem. Biophys. Res. Commun..

